# A Physical Adaptive Material Motor Unit Neural Network: A Hygromorph Composite Material Machine

**DOI:** 10.1002/advs.77473

**Published:** 2026-09-06

**Authors:** Charles de Kergariou, Helmut Hauser, David Correa

**Affiliations:** ^1^ Bristol Composites Institute, School of Civil, Aerospace and Mechanical Engineering University of Bristol Bristol UK; ^2^ School of Engineering Mathematics and Technology University of Bristol Bristol UK; ^3^ Bristol Robotics Laboratory Bristol UK; ^4^ School of Architecture University of Waterloo Cambridge Ontario Canada

**Keywords:** 4D printing, biocomposites, physical neural network, physics‐aware back‐propagation, shading facade

## Abstract

Advances in novel materials science enable structures to function as intelligent machines by embedding memory and learning capabilities directly into materials. Our work introduces a physical adaptive material motor unit neural network, leveraging a new generation of controllable actuators composed of wood‐ and carbon black‐based composites, sensitive to temperature and relative humidity. This structure constitutes a first step toward the objective of embodying learning capability into materials. These material actuators are assembled into a motor unit‐like structure inspired by muscle contraction trigger, forming an intelligent machine capable of dynamic shading control that can be used, for example, in buildings. The machine is governed by a neural network trained on over 350 experimental data points collected under diverse environmental conditions. By establishing a new data‐aware backpropagation training, we show that the machine predicts shading responses and learns, via an artificial neural network‐based surrogate model, to predict appropriate behavior incrementally as the database expands. We also demonstrate the ability of the machine to optimise configurations to achieve similar shading outputs under two distinct conditions.

## Graphical abstract

This study introduces a new class of material‐based intelligent machines. It presents a Physical Adaptive Material Motor Unit Neural Network, an assembly of smart 4D printed actuators and a controlling system that adapt through environmental interaction its shading behavior. The material‐based machine regulates light through a self‐actuating facade, which opens in response to temperature and humidity.

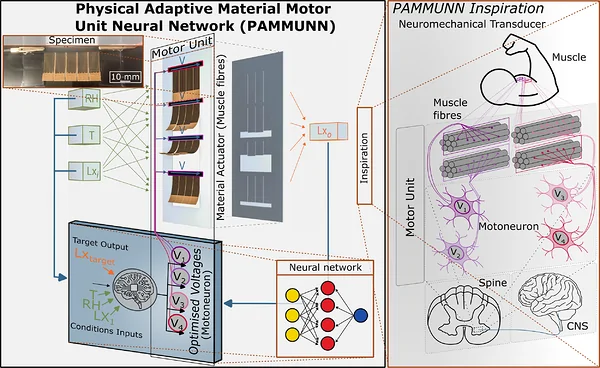

## 1 Introduction

Material actuators are increasingly designed following nature‐inspired mechanisms [1, 2] that enable the use of available energy in the environment to create motion [3]. The material within the actuator stores energy [4] and turns environmental stimuli, such as humidity change, into mechanical energy [5]. Materials that are humidity‐sensitive are called hygromorphs [3]. Other material actuators have been developed to achieve motion in response to environmental stimuli, such as change in temperature [6, 7], humidity [5] or pH [8]. Such energy transformation is used to create shading structures in construction [3] that adapt to sun angles, smart clothes [9], smart biomedical implants [10] or sensors that provide a kinetic response to environmental changes [11]. However, three key aspects hinder the widespread adoption of technologies based on material actuation, in particular for temperature/humidity triggers. The first challenge is the reduced scalability of the output of the actuation, such as amplitude [10, 12] and load carried [13]. This is still a problem, despite the existence of large printing/additive manufacturing capabilities [14]. The second problem is the lack of precise control of the output (e.g., actuating amplitude) for the wide variety of environmental conditions such actuators will be subjected to [15]. In situ control is one of the main challenges facing 4D printing technology on its way to industrial applications [10]. A third challenge is their partial irreversibility, which complicates precise actuation control [16].

**FIGURE 1:**
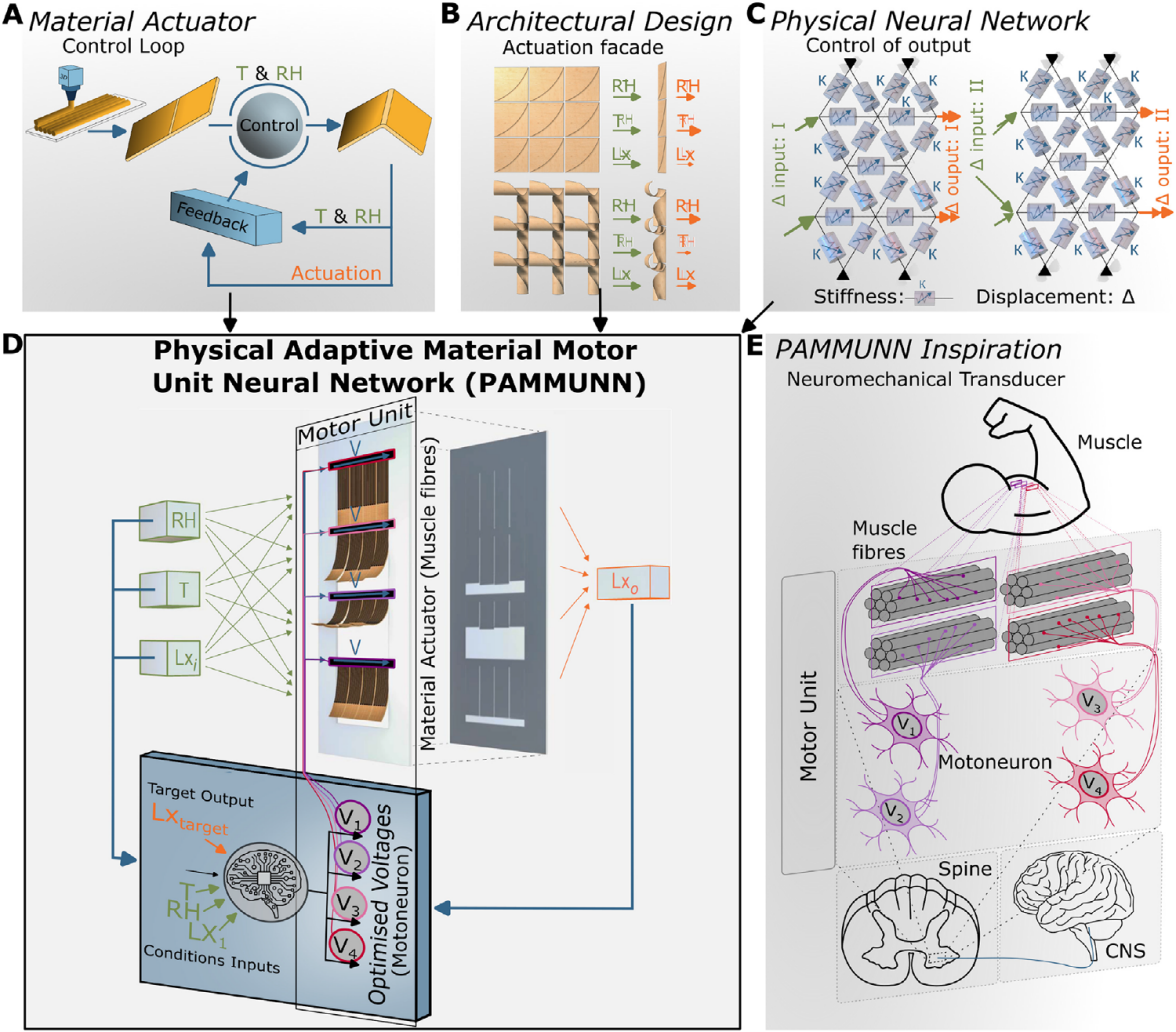
Concept and inspiration of the physical adaptive material motor unit neural network. Three main types of technology are combined here to conceptualise PAMNN: (A) Material Actuation [5, 19] and its control [20, 21], (B) a self‐shading Architectural Design [3, 22] and (C) a Physical Neural Network [23, 24] with the schematic of a Mechanical Neural Network by Lee et al. [25]. (D) The PAMNN‐inspired structure of this study consists of four humidity‐triggered actuators, whose deformation is controlled by heating a conductive composite. The structure imitates a one layer NN which turns three inputs (T: Temperature, RH: Relative Humidity, ${\mathrm{Lx}}_{i}$: Input light) into an output (${\mathrm{Lx}}_{o}$: Output light), which is represented by the light inside the box covered by the actuators. Due to its NN‐based digital training, the output of the structure is predicted in a similar way to a feed‐forward neural network, with voltage serving as control of the opening of the neurons. The structure retains actuation data, enabling it to continuously learn from the interactions between inputs and outputs. (E) Inspiration for PAMMUNN in which the Central Nervous System (CNS) creates the inputs to control the force to generate movement [26] via the rate of actuation potential firing rate in the neurone [27]. The information is transmitted through the spine to the motor units, which are neuromechanical transducers made of a motor neuron and the muscle fibers it triggers for contraction.

One way of solving the limited size of actuators is to split them in small sub‐units assembled in arrays, whose output is the cumulative effect of all individual actuators. For instance, that is the concept behind self‐shading facades in construction engineering [3, 17]. However, programming an adaptive facade with material actuators in a real building involves the interaction of a virtually infinite number of micro‐climatic environments [18]. This involves differences in sun exposure, height (i.e., air is more humid near ground than at the top), temperature, proximity to vegetation, partial shading from other buildings, and interaction between actuators. A top‐down approach to programming such a system is practically not feasible, due to significant computational costs involved in predicting all possible combinations of conditions for each local situation. To account for the complexity of the environment, the material actuator itself should *remember* and *process* information to adapt to the local conditions and give the required output. Hence, one layer of such actuators has the potential of behaving similarly to a layer of a neural network, with each actuator being a node. Structures with multiple small actuators provide an opportunity for fine control of the output. Figure 1 presents the sources of inspiration for the proposed artificial neural network machine made with material actuators, which we call a Physical Adaptive Material Motor Unit Neural Network (PAMMUNN).

Proposed concepts of Physical Neural Networks [23, 25, 28, 29] possess architectures, in which connections between artificial neurons are stiffness‐based and make use of concentrated parameter elements [30]. Physical neural networks also feature topologies made of assemblies of connecting nodes, each of them autonomous from an energy perspective. The use of materials with intrinsic actuation capabilities is a first step to provide true autonomy. This aligns with the principles of *physical control* [31] and *morphological computation* [32], leveraging oscillations, sequences, and reactions for self‐regulation. For instance, implemented with compact, autonomous robotic systems [4, 33], and also actuating facades and architectural designs [34, 35]. Physical Adaptive Material Neural Networks, the actuation adaptation of the Engineering Material Neural Networks concept [36], embody three key design principles: i) be made of material actuating neuronal nodes; ii) be able to find optimal actuating configurations to reach targeted outputs from given inputs; and iii) demonstrate learning at the material actuation level to boost reversibility and adaptability. The ultimate goal of the development of physical neural networks is to create a machine with no traditional computing hardware (e.g., GPUs) to achieve the three design principles mentioned above [23].

The physical adaptive material motor unit neural network (PAMMUNN) we propose in this work is a step toward the development of physical adaptive neural networks with actuation, energy autonomy and self‐regulation not yet fully at the intrinsic material level. As described in Figure 1D,E, the PAMMUNN is inspired by a biological motor unit (combination of a motoneuron and the muscle fibers whose contraction it controls). Similar to some biological systems, the PAMMUNN presented in this study is capable of receiving environmental inputs to control shading output. To attain this performance, the design of this PAMMUNN is based on the use of natural fibers reinforced polymer composites, which are responsive to humidity [13]. As a consequence, we define a new humidity‐responsive (hygromorph) composite architecture based on combinations of wood/thermoplastic and carbon black/PLA filaments deposited using filament‐fused fabrication. The wood fiber‐dominated actuation [5, 37] of this adaptive material is controlled via electrical power heating [19, 38]. With this material, we create an assembly of four identical actuating blocks which are equivalent to the nodes of a neural network capable of controlling the penetrating light (Figure 1D). Physical training in biological motor units adapts the firing rate of the actuation potential and synchronization of the motor units [27, 39]. Like in motor units, the PAMMUNN has its mechanical actuation controlled via electric voltage by a centralized neural network [26]. We also employ physical training, allowing the PAMMUNN structure to learn adaptive behaviors through iterative physical updates. To do so, a physics‐aware back‐propagation‐inspired training is developed by linking inputs (temperature, humidity, environmental light) to the output light by controlling the heating of the material actuators. PAMMUNNs are intermediary steps to the creation of engineering material neural networks, hence, they do not present all the characteristics highlighted for physical neural networks [36]. For instance, it learns with computation and not intrinsically within the material properties, then transmits the acquired knowledge to the physical 4D printed system. We also demonstrate that this PAMMUNN biocomposite hygromorph composite machine learns its behavior through data acquisition.

## 2 Results

### 2.1 PAMMUNN Using Data‐Aware Back‐Propagation Training

#### 2.1.1 Database: Acquisition

**FIGURE 2:**
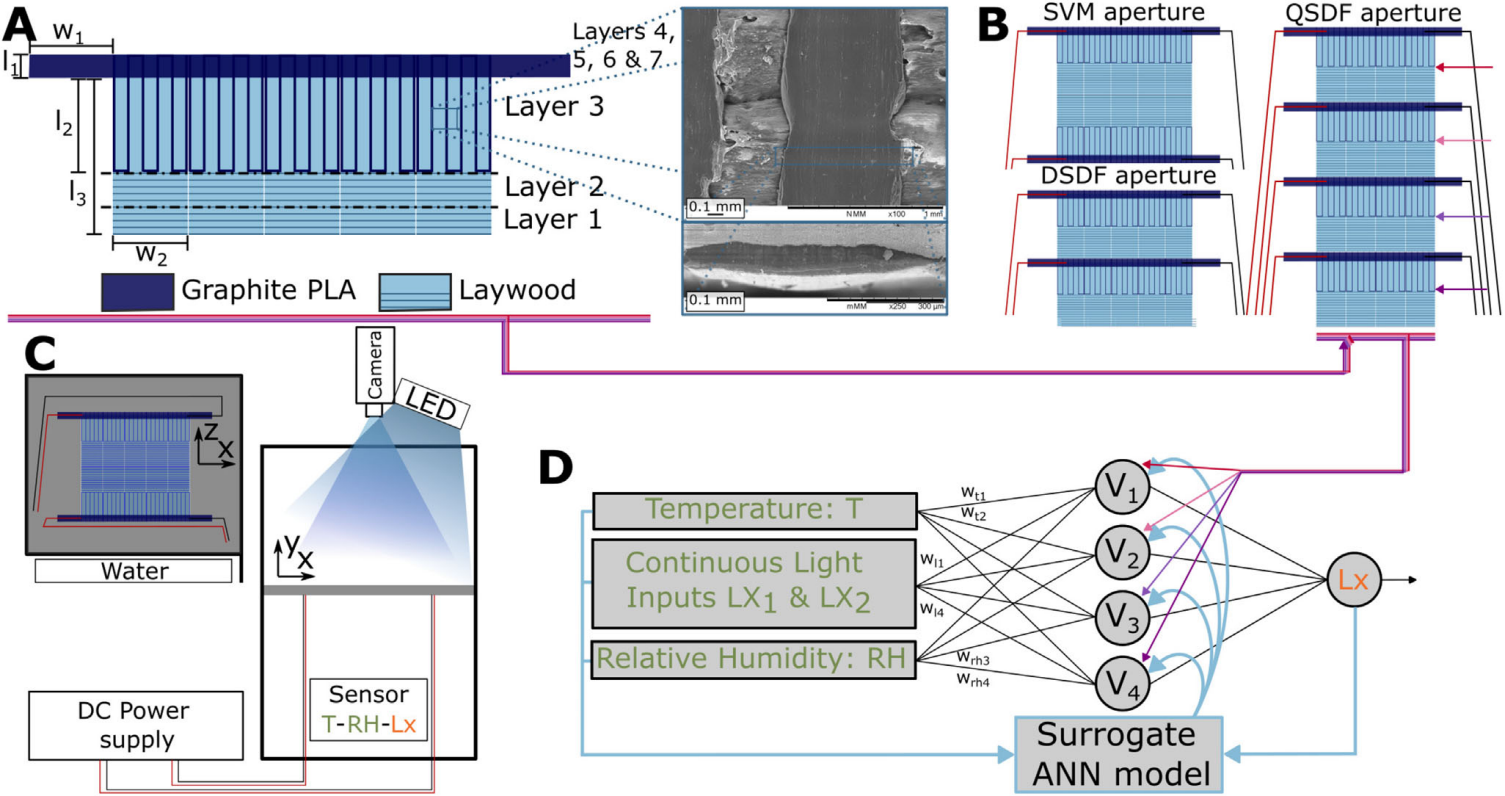
Printed geometry, stacking sequence, SEM images, spatial architecture of the specimens fabricated and experimental setup implemented. Definition of speed reversibility and actuation strain. (A) Left schematic: Schematic of the stacking sequence of the specimen Right images: The top image is a top view SEM photo of the junction between the natural fiber and graphite‐filled PLA. (B) Spatial architecture of the specimens for different tests named Symmetrical Vertically Mirrored (SVM) aperture, Double Stacked Down Facing (DSDF) aperture and Quadruple Stacked Down Facing (QSDF) aperture. The red and black lines on each side of the specimens are the electric‐connector to the DC power supply presented in Figure 2C. (C) Schematics of how the SVM aperture specimen of Figure 2B is positioned. (D) Schematic of the PAMMUNN for the QSDF configuration of actuators.

**FIGURE 3:**
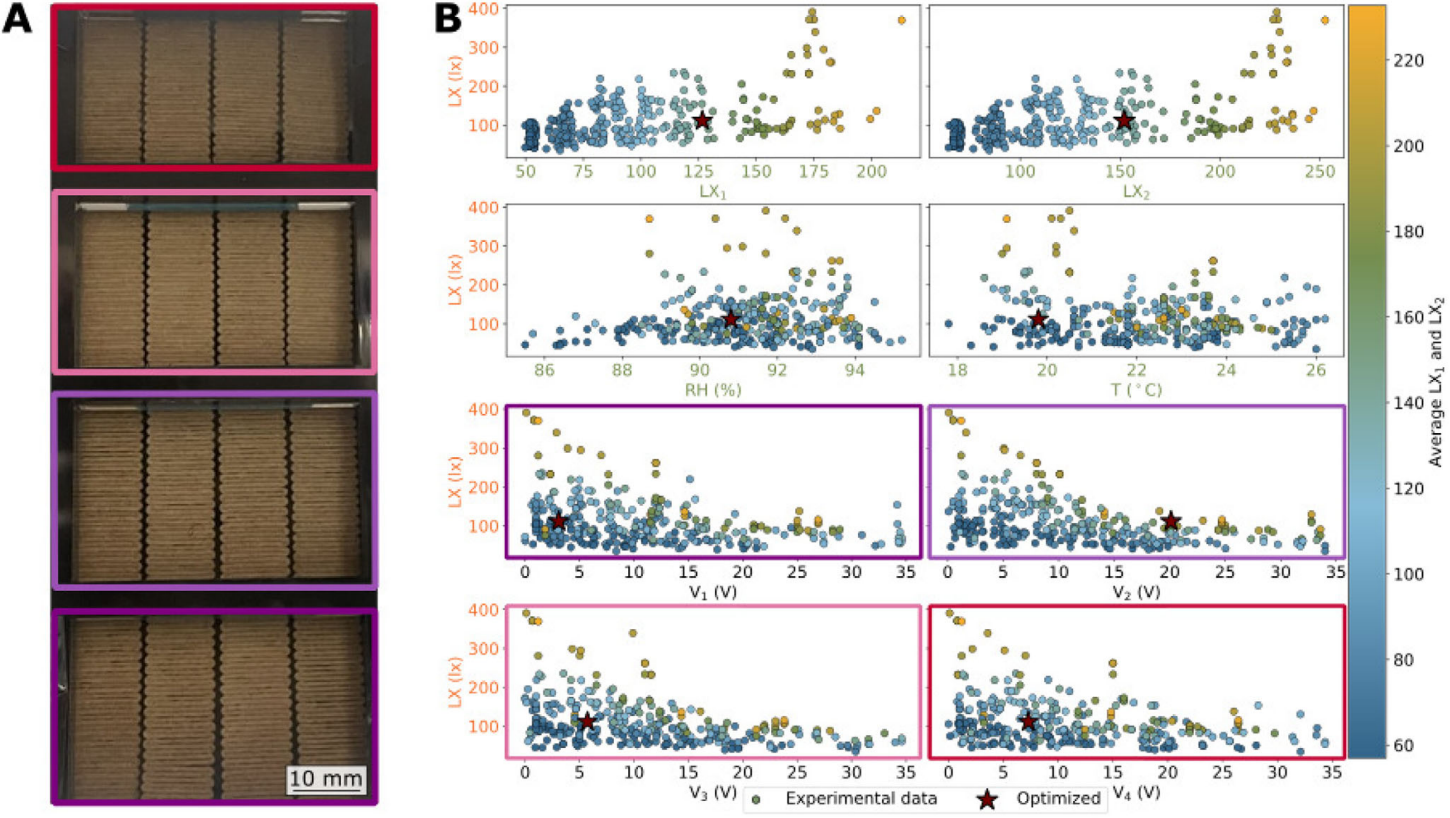
Example of database used for scenarios 1 and 2. (A) Image of the actuating structure for the 4*1 down actuation setup. (B) Database created from testing the QSDF aperture set up from Figure 2. Black stars are examples of optimized input voltage via the Neural Network control system presented in Figure S2.

The first PAMMUNN prototype was created by assembling four novel hygromorphic actuators side by side to form a small‐scale shading structure (see Methods section). This assembly of soft robotic actuators is called Quadruple Stacked Down Facing QSDF aperture setup (Figure 2), and it was defined after initial study presented in Supporting Information. In the present paper, *structure* refers to the assembly of hygromorphic actuators and the DC power supply connected with them to control their actuation, and *machine* refers to the entire system with control. Figure 3B presents an example of training database used for the model in Figure 2D. Such database was used to train a data‐aware back‐propagation training to control the shading of PAMMUNN. We define in the method section data‐aware back‐propagation training as a new type of back propagation training method for physical neural network functioning in a similar manner to physics‐aware back‐propagation trainings [23] without the need for a digital model.

Figure 3B shows the impact of different parameters on the output of the system (light going through). The input lights ${\mathrm{LX}}_{1}$ and ${\mathrm{LX}}_{2}$ show a strong correlation with the output light. In general, the larger the light input, the higher the light output. The temperature and humidity do not show a clear correlation with the output of the structure in Figure 3B. Without pre‐constraining, biocomposite‐based actuators tend to bend with the increase of humidity [3, 15]. While humidity influences actuation, voltage dominates this effect. The observed humidity range remains narrow (Maximum range between [85%; 95%] as seen in Figure 3B) compared to the typical actuation ranges (Several tens of relative humidity percentages, the minimum range observed by a previous review [37] is 40%) for such soft robotic materials. The scatter of the data related to the four input voltages shows that the larger the voltage input, the lower the output light.

#### 2.1.2 PAMMUNN: Implementation

We present scenarios to explore the ability of the optimization technique and the performance of the physical adaptive material neural network machine. In both scenarios 1 and 2, the time scale of evolution is in minutes, which indicates a slow change of light output. However, in potential applications the small variations in lights from the sun during the day would induce slow continuous changes in light input that the system would be able to follow at its speed. Slow changes in light outputs also permit to limit the impact of the lone cloud perturbations. If faster changes of light output is required, such as demands from the operator, then the overshoot strategy can be amplified to achieve quicker convergence.

**Scenario 1**

In this scenario the light output is set to converge out of an interval of light into another one when the user decides to change the target desired light of the system. The optimized voltage values obtained from the data‐aware back‐propagation training drive the system to the target light interval, demonstrating how a self‐shading structure dynamically adjusts its shading output based on operator input.

**FIGURE 4:**
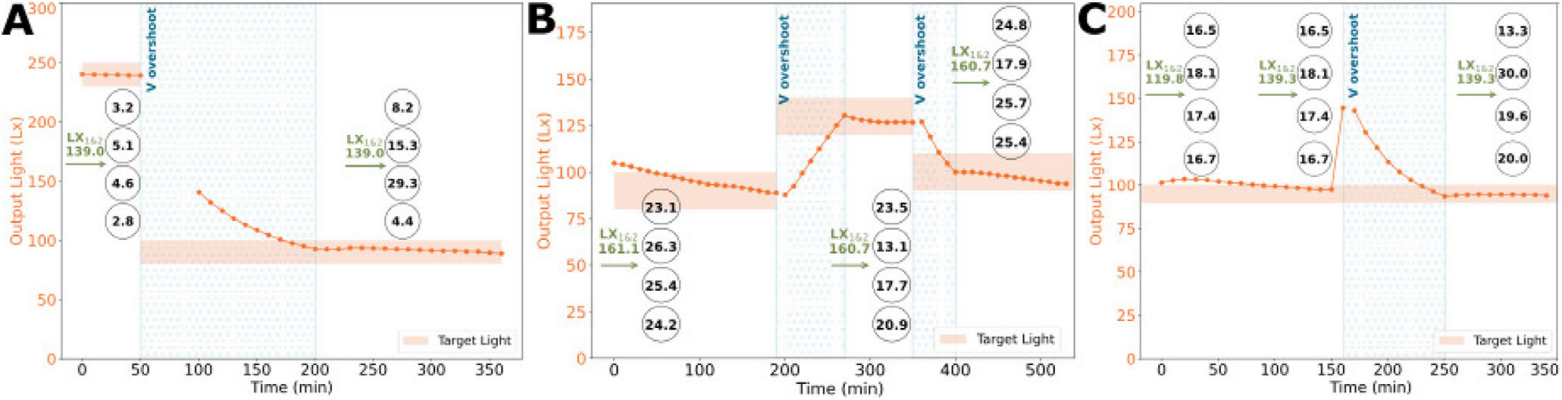
Example of output light evolutions for scenarios 1 and 2. (A_) Scenario 1: Example of one stage convergence obtained for the 4*1 down actuation setup. The blue circle filled section corresponds to the time in which the optimized voltages are artificially increased to reach the predicted output value faster. The orange bands show the range for the target output light intensity. The black circles present values of the voltage induced in each individual actuating specimen. The top value is related to the top actuating specimen presented in Figure 3A and to the V1 values of Figure 2D. The bottom value is related to the bottom specimen shown in Figure 3A and the V4 value of Figure 2D. (B) shows an example configuration for the scenario 1 with multi stages of optimization. (C) Example configuration for scenario 2.

In the configuration presented in Figure 4A, the input light is 139.0 (average between ${\mathrm{LX}}_{1}$ and ${\mathrm{LX}}_{2}$). The target output light (orange bands) after decision of the operator ranges from 80 to 100 Lx. The voltage inputs initially are $V_{1}$ = 3.2 V, $V_{2}$ = 5.1 V, $V_{3}$ = 4.6 V and $V_{4}$ = 2.8 V (left circles in Figure 4A). Due to this range of input voltage, the output light initially converges toward 239.4 lx (orange line between 0 and 50 min). Then, the neural network lowers the output light to 88.8 Lx, resulting from a new set of voltage values ($V_{1}$ = 8.2 V, $V_{2}$ = 15.3 V, $V_{3}$ = 29.3 V and $V_{4}$ = 4.4 V). The converged output light values show a 150.6 Lx gap—from the initial 239.4 Lx to the final 88.8 Lx in the targeted interval. This 150.6 Lx gap closed in under 150 min (blue circle region). The second configuration presented for this scenario (Figure 4B) aims to show that the machine can adapt to smaller and multiple changes of targeted light outputs by the user. Figure 4B demonstrates that the neural network‐like physical machine reliably converges multiple times to the different target light output. Four additional example of this scenario are given in the Supporting Information (see Figures S2–S5).

**Scenario 2**

In this scenario, once convergence is achieved in the target light interval, the light input is altered—and the system re‐converges to the original target interval between 80 to 100 Lx (orange band in Figure 4C). This scenario demonstrates how the system adapts autonomously when the input light changes—such as shifts in the sun's position relative to the shading structure. Figure 4C illustrates a scenario where, after converging to the 80–100 Lx target interval, the input light suddenly spikes—from 119.8 to 139.3 Lx (average of LX1 and LX2). In this configuration, the QSDF aperture restores the output light to the target interval in under 70 min, achieving a 50 Lx reduction. Three other examples of this scenario are presented in Supporting Information (see Figures S6–S8).

#### 2.1.3 PAMMUNN: Incremental Learning

**FIGURE 5:**
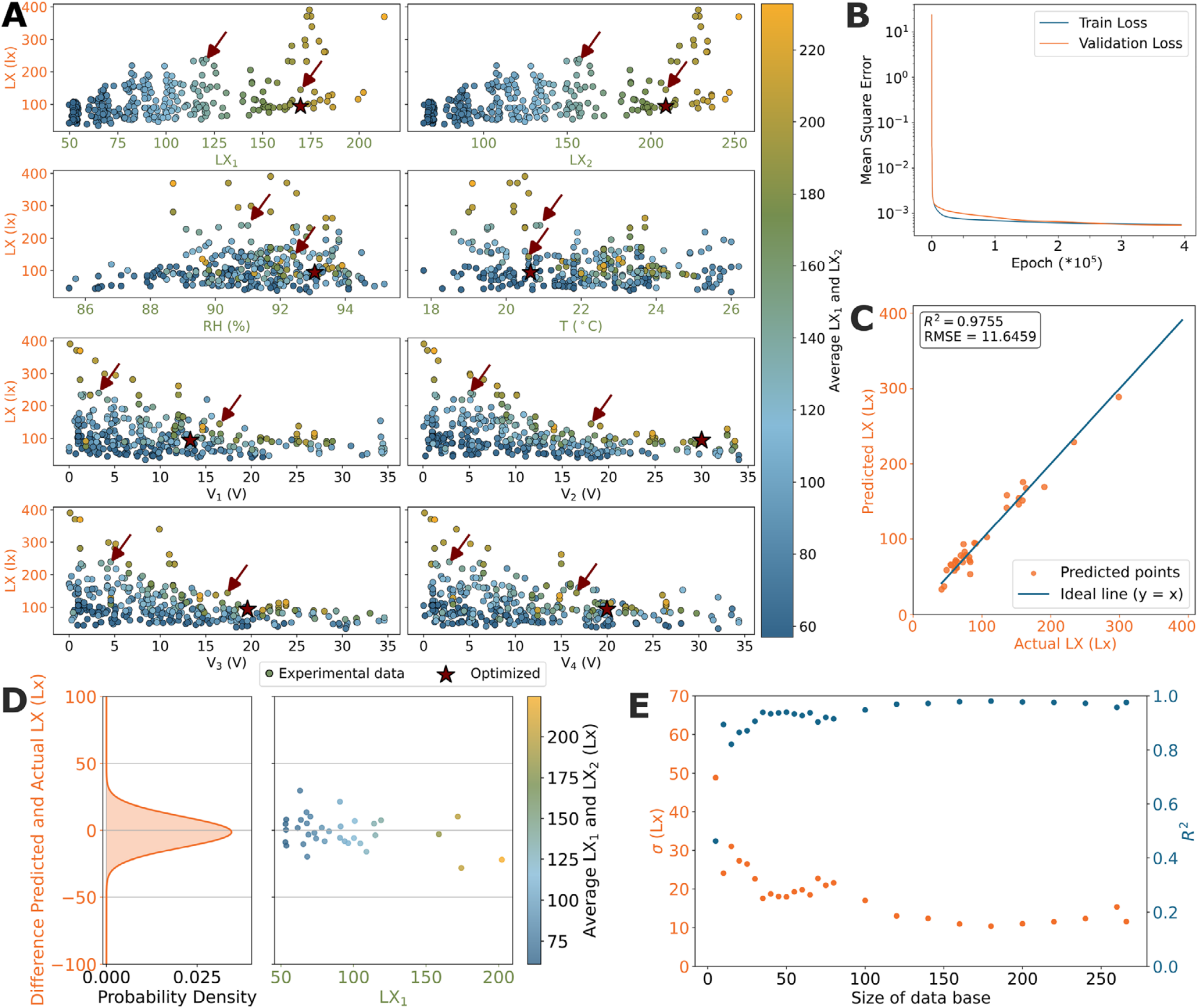
Optimization and results for incremental learning demonstration. (A) Shows the database used to calculate the optimal voltages to reach the target interval. (B) Evolution of the Error during training of the neural network with all the database considered. (C) Actual versus Predicted data for the validation data used during training of the last iteration of the database. (D) Gaussian distribution of the difference between the actual and predicted LX values for the validation data at the last iteration of the training. (E) Evolution of the standard deviation of the difference between predicted and actual values and of the coefficient of determination for every iteration of the training during demonstration of incremental learning for the machine created.

In Figure 5A, the downward red arrows indicate new data points added to the database, compared to the initial scenario in Figure 3B. These arrows show the increase of database size for this new optimization case potentially inducing an incremental learning for the machine. Figure 5 shows the different steps of the database training during demonstration of incremental learning. This test aims to present how an incremental increase in database size improves the quality of the prediction by the data‐aware back‐propagation training. The database increase incrementally in size as the machine operates it collected data of the relation between input and outputs parameters. Figure 5B shows a sample convergence curve for the proposed model. No increase in the validation error was observed in the error versus epoch plots highlighting the absence of overfitting during training. 50 evaluation points were used in the data‐aware back‐propagation training. Figure 5C compares the actual vs. predicted values, evaluating the performance of the trained neural network on these points of the database. From this data, the errors between actual and predicted values were calculated and plotted, with their Gaussian distribution shown in Figure 5D. Two metrics were used to evaluate the machine's adaptability, the coefficient of determination ($R^{2}$) and the standard deviation σ of the related Gaussian distribution of the difference between actual and predicted values used in the neural network of the data‐aware backpropagation training (Figure 5C). The two metrics are plotted against the size of the database in Figure 5E.

Figure 5E demonstrates that the standard deviation of prediction errors drops sharply as the database grows to 120 points, then stabilizes during the last validation to ≈10 Lx. In Figure 5E, the coefficient of determination rises steeply up to 120 database points, then gradually converges to ≈0.98. From the first set of data ($R^{2}$=0.46), to the last iteration with the full database ($R^{2}$=0.98), the coefficient of determination representing the efficiency of the PAMMUNN to find the optimal configuration to achieve its targeted output increases by 113%. These two evolutions highlight the learning ability of the machine. As the database increases in size the differences between the predicted and the actual values obtained reduces leading a better prediction ability of the machine. For instance, the Gaussian distribution of the difference between predicted and actual values shown in Figure 5D reveals that four in five predicted values falls within a ±14.9 Lx range of the target. This range reduces in size along with the addition of tested data points proving the machine's adaptation ability. The parameters displayed in Figure 5E ($R^{2}$ and σ) prove the convergence of the model after 120–150 data points. Both parameters do not display significant improvement after 150 data points, showing that the model is both precise in predicting the values ($R^{2}$ ≈ 1.0) and able to predict the required behavior in repeatable manner across the database analysed (σ constant).

### 2.2 PAMMUNN: Physical‐Mode

**FIGURE 6:**
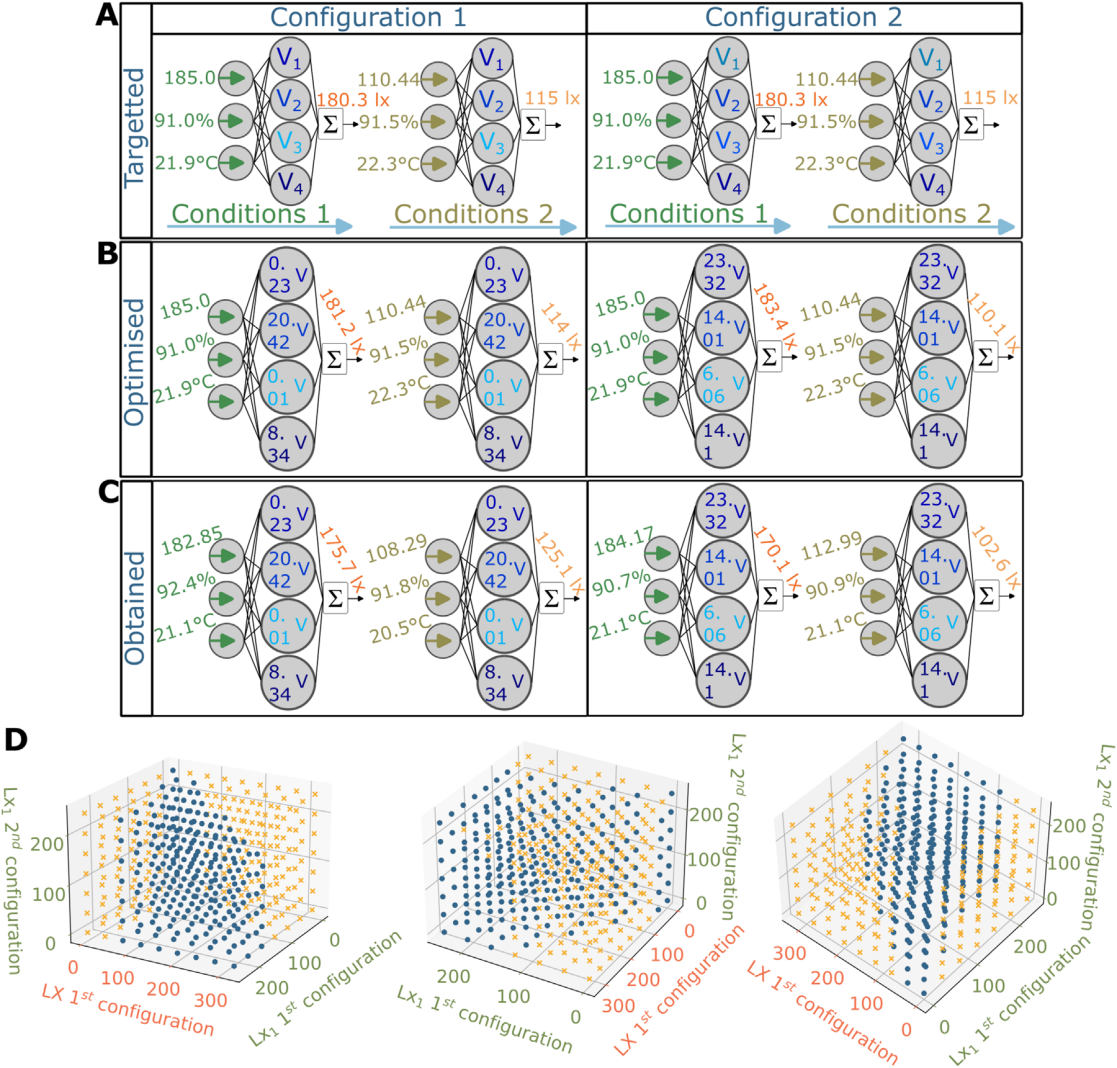
Example of physical‐mode operating. (A) Targeted output for given conditions. (B) The optimized voltages are obtained from the specified conditions and targetted outputs. Due to the structure's physical constraints, only certain output values are attainable. To this extend, the optimized output values differ from the targeted ones in (A). (C) The optimized voltages previously calculated are implemented in the physical structure to obtain outputs close to the ones optimized. (D) Design space for the physical‐mode of the machine (three different angles of view). Blue dots corresponds to the configurations and conditions for which the shading structure developed found at least two sets voltages for the two targetted outputs given two inputs. This is a necessary condition to allow the setup to be used in this mode. The yellow crosses correspond to the configurations and targetted output for which the shading set up could not find two sets voltages allowing for the machine to function in physical‐mode.

Inspired by the neural network tests performed by Lee et al. [25], Figure 6A–C presents the same input under two different structural configurations. Additional test results of the same nature are provided in Figures S9 and S10. This physical‐mode was implemented and tested with the conditions presented in Figure 6. Two other examples are presented in Supporting Information.

This figure shows that a difference exists between optimized output values in Figure 6B and those physically obtained in Figure 6C. These differences are 5.5, 11.1, 13.3, and 7.5 Lx for the configuration 1 with conditions 1 and 2, and configuration 2 for condition 1 and 2, respectively. Two main reasons for these differences are the variabilities observed in the physical systems of the scenarios presented in the previous sections (see Figure 4 and the standard deviation introduced in Figure 5). The second reason is the difference in terms of temperature, light and humidity observed between the two types of datasets, Optimized (Figure 6B) and Obtained (Figure 6C). For example, in configuration 2 under condition 1 (Figure 6C), both light and humidity fall below the optimized levels shown in Figure 6B. This artificially reduces the output, as reflected in the experimental data.

Figure 6D shows the design space of the physical machine behaving as in the physical‐mode. Lower light input in one configuration limits the use of physical‐mode, reducing viable options of sets of voltage to obtain the second configuration. This partly explains the difference of outputs between targetted values observed in Figure 6A and the optimized one in Figure 6B. For instance, in configuration 2 condition 1 the optimiser modified the output light from 180.3 to 183.4 lx to be able to fins a configuration able to come close to the required input and targetted outputs. However, in Figure 6D as the input light of the first configuration increase, proportion of second configuration achievable increases as well. This is explained by the fact that low sets of “low” voltages will allows increasing number of large light input and, the sets of “high” voltages will permit low light outputs to be obtained. For similar reasons a limited number of second configuration outputs are available when the output light of the first configuration is low. Low outputs required for the first configuration limits the possibility of using the physical configuration in physical‐mode as it implies that one of the conditions will have low light inputs. It could also be due to the fact that at low output for the first configuration, large voltages at all neurons limit the adaptability of the soft robotic material for the second configuration. This dariability is important in the context of this study as we proposed a 5 V mandatory difference between at least 5 of the voltages of the two configurations to display the physical‐mode possibility of the machine. Expanding the actuating structure via addition of more openings enlarges the design space, enabling more aperture variations per input‐output requirement.

## 3 Conclusion

This study investigates a physical actuator architecture made of adaptive biocomposite materials that approximates neural network behavior. A new material actuator was designed using a wood‐based thermoplastic and conductive carbon black‐reinforced PLA, and a novel geometry was defined for shading structures. The biocomposite actuator, with its fiber‐dominated actuation structure, is activated by drying the fibers through heating from the conductive material. Initially, two actuating configurations were compared, showing that downward‐oriented specimens are preferable for creating self‐shading structures than configurations with both upward and downward actuators. The structure designed for this study therefore includes four identical, parallel actuator apertures for shading. The shading output was generated by controlling voltage through the carbon‐black reinforced PLA section, using environmental inputs like temperature, humidity and light. The light output of the adaptive material actuator was controlled using a newly defined *Data‐Aware Back‐Propagation Training*, providing the first self‐learning control system for material actuators, to the best of the authors' knowledge. This training was conducted on an experimentally acquired database, which at the end of the study comprised more than 350 points. A neural network trained with the new Data‐Aware Back‐Propagation provided the optimized control voltages. Neural network validation confirmed that four of five optimized voltages kept the light output within ±14.90 Lx. The hygromorph machine's efficiency was tested in two scenarios, demonstrating the ability of the control technique to manage light output, whether the target changes or inputs vary. In these scenarios, the objective light was achieved in a repeatable manner within a 20 Lx interval. An overshoot voltage strategy was also employed to accelerate actuation of the structure compared to conventional hygromorphic structures. We were able to display incremental learning by increasing the size of the database as the machine operates.

The Physical Adaptive Material Motor Unit Neural Network machine consists of actuating material neural nodes. It optimizes configurations from inputs and demonstrates learning digitally, not at the material level, as the final coefficient of determination for the actual to predicted light value was 0.98 for the final database acquired. The Data‐Aware Back‐Propagation Trained self‐trained PAMMUNN also achieves two optimal configurations that convert two different inputs into similar outputs, proving ability to function as a neural network. In this mode, PAMMUNN reduces reliance on external digital and/or electronic systems, enabling future permanent learning based on the actuating material's physical properties.

## 4 Methods

### 4.1 Printing and Prototype

The specimens were produced with laywood meta 5 (from Kai Parthy) and conductive carbon black‐reinforced PLA (from Protopasta) with the stacking presented in Figure 2A. The influence of humidity on the properties of laywood material has been studied in previous studies [5], for instance, its stiffness ranges from 37.51 ± 4.27 to 1446.20 ± 189.80 MPa depending on the conditioning Relative Humidity (RH). Krüger et al. presented the shrinkage of laywood meta5 to be 2.6% from 100% RH to 80% RH and 3.5% from 100% RH to 30% [40]. Sahin et al. further detailed the moisture expansion of the material depending on its printed pattern [41]. The thermal, mechanical and electrical properties of conductive carbon black‐reinforced PLA are presented by Dezaki et al. [42]. For instance, relative to room temperature the temperature coefficient of resistance was 1.38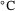 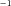. Layers 1 and 2 were made of unidirectional printing laywood. Layer 3 had also an unidirectional printing path but with a filling of 20% and printed with Protopasta graphite reinforced PLA. Layers 4–7 were made with rectilinear printing paths of Protopasta carbon black PLA. The dimensions presented in Figure 2A for the specimens are as follow: $w_{1}$: 9.5 mm, $w_{2}$: 9.5 mm, $l_{1}$: 5 mm, $l_{2}$: 22 mm and $l_{3}$: 30 mm. The composites were printed using a Prusa MK4 at a printing bed temperature of 80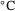 and a nozzle temperature of 220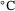. The heights of the layers (0.4 mm between laywood layers and 0.2 mm between laywood and PLA layers) were chosen to obtain a good contact between the two different soft robotic materials (Figure 2A). The contact between the different layers of an actuators was key to control actuation and limit irreversibility [16]. Hence, the bottom image was an SEM photo of the cross section of the contact between the laywood meta 5 material and the carbon black PLA. The specimens presented in Figure 2B were then attached to a black laser cut piece of Perspex with size of holes adjusted to the size and distribution of the specimens created to let as much light go through as possible when the actuators were curved. Figure 2B shows the different positioning of the specimens evaluated. SVM aperture: Initial test with symmetric specimens. The top specimen was orientated downward and the bottom specimen upward. DSDF aperture: second test with both specimens orientated downward. QSDF aperture: Initial geometries for Material Neural Network‐like control of light. For the specimens presented in Figure 2B SVM aperture and DSDF aperture, one hole 50 mm wide and 50 mm high at the centre of the black perspex plate was cut. For the specimens presented in Figure 2B QSDF aperture, four holes of 40 mm wide and 25 mm high were cut, instead. For the specimens used in this last structure, the number of individual soft robotic material actuators was reduced from five (Figure 2A) to four. The specimens were fixed with an electrical clamp connected to a DC power supply and attached to the conditioning box via magnets.

### 4.2 Validation of the Conditioning Setup

Figure 2C shows a schematic of the set‐up developed to record the actuation of the specimens in Figure 2A. The specimens were inserted into a box made of perspex and completely opaque, apart from the transparent perspex face in front of the bowens LPD1 LED light panel and the Allied Vision Alvium 1800 U‐234m camera. In the 55 mm‐high box, a floor was positioned at 40 mm, beneath which two large Petri dishes filled with deionized water were placed. The humid air rose to the upper floor through square openings in the floor, where the light, humidity and temperature sensors were positioned. The temperature and relative humidity level inputs will be referred in green throughout this paper, outside of the introduction. The light measured with the sensor will be considered as the output of the test and is highlighted in orange throughout the rest of this paper. Initial trials were carried out to verify the humidity inside the conditioning boxes. These trials confirmed that relative humidity stabilized in the conditioning boxes after 24 h. As a result, all recordings began only after a minimum 24‐h waiting period following box closure.

**FIGURE 7:**
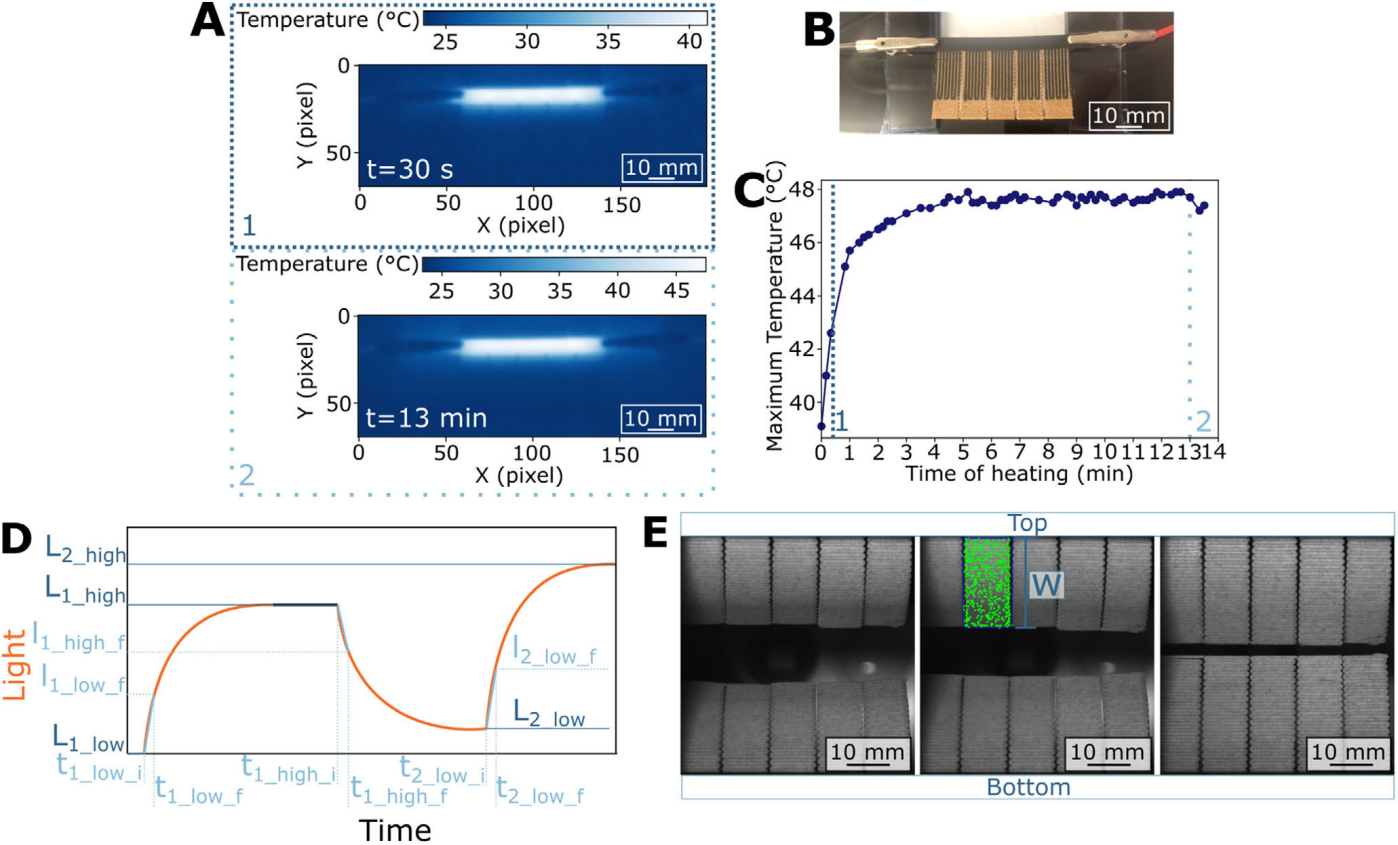
Thermal test of the 4D printed actuators and definition of speed reversibility and actuation strain. (A) Example of thermal images recorded during the heating of one specimen. Top and Bottom images are recorded after 10 s and 13 min of heating, respectively. (B) Image of the 4D printed specimen heated by setting up the DC power supply with a 50.0 V input to record thermal images. (C) Evolution of the maximum temperature with time when the specimen is heated with a 50.0 V input in the DC power supply. (D) Definition of reversibility and speed parameters for soft robotic material actuation. (E) Example of images recorded during experiment testing SVM aperture, after the voltage is set up to 50.0 V.

The heating generated via the DC power supply shown in Figure 2C is detailed in Figure 7A,B. Thermal images were obtained by positioning the specimens described in Figure 2A in the box set up. A Flir A70 thermal camera was used to record images while the specimen was being heated. Images in Figure 7A show the heat map of the specimen in Figure 7B after 10 s and 13 min of heating with the DC power supply set up at 50.0 V on input. Figure 7C shows the evolution of the maximum temperature on the surface of the specimen during heating. The temperature reaches a stable value after 5 min. The thermal images show constant values along the surface of the carbon black/PLA composite, showing no large defects present in the printed material.

The actuator was controlled by setting up voltages ranging from 0 to 35 V through the conductive material. The current going through the conductive material locally heats up the actuators as shown in Figure 7A. This dries the humidity responsive material leading to the flattening of the actuator. The functioning of one actuator is presented in Figure S1G. It highlights the correlation between the voltage set to an actuator and the surrounding environment. For instance, it shows that increasing the applied voltage increases the measured humidity. Therefore, the actuators not in immediate proximity to the conductive composite with the voltage applied tend to bend more and then let more light go through the structure whereas the two humidity responsive material in close proximity to the voltages are dried and therefore tend to close, letting less light pass through. These complex interactions among parameters was the reason for creating the data‐aware back propagation training to control the machine.

### 4.3 Design of the Material Actuating Neurone

Since this was the first time the combination of material was 3D printed, the initial design required optimization based on quantitative experimental measurements. For this reason, the performance of the two series of specimens in Figure 2B (SVM aperture and DSDF aperture) has been compared for benchmark purposes. The specimens were positioned inside the testing box, then voltage inputs were alternated between 0.0 V during at least 58 h and 50.0 V for 10 h. Light, temperature and humidity inside the box and images of the actuators were detected using the experimental setup previously described in Figure 2C.

In the present context, reversibility is the capability of an actuation mechanism to obtain similar light changes between two actuator positions independently of the history of the actuator [37]. This reversibility relates to the ability of the actuator to repeat actuation amplitude. Every reversibility data for hygromorph must be associated with an actuation history [37]. This history is presented through a set of parameters, like the relative humidity variation $n_{1}$, the number of repeats $n_{2}$ and the duration of the conditioning $n_{3}$, the latter being defined as the difference between the actuation trigger start and the convergence value as presented in a previous publication [37]. In the present study, the relative humidity is the main source of actuation, but the actuation trigger is the voltage. Therefore, the reversibility parameter $n_{1}$ is represented by the variation of the voltage. As a consequence, the parameters in the present study are $n_{1}$ = 50 V, $n_{2}$ = 6 and $n_{3}$ = 10 h. To measure the effective reversibility of the structure, the light recorded by the light sensor serves as output. The term $\Theta_{N}$ in Equation 1 provides the average of light output amplitude variation obtained over a given number of repeats.

(1) $$\Theta_{N}=\frac{\sum_{n=1}^{N}\delta Lx}{N}$$

In Equation (1), δLx is the variation in light of the soft robotic material actuators between opened (with voltage off) and closed (with voltage on) configurations. *N* is the number of iterations conducted during the reversibility test. The actuation speed of the material structure—here, the rate of light change—can be determined by two distinct properties [37]: time to convergence, and time derivative of the output parameter. The time derivative of the output parameter is defined in the present study as the light variation, light varying speed, ($\xi_{l}$ in Equation 2) immediately after turning on or off the voltage. The time to converge of the material actuating structure is defined as the time taken to reach 95% of the final light output. For instance, in Figure 7D this time corresponds to the first time when the light reaches inside the interval [0.95; 1.05]*$L_{1\_high}$ and is defined as $\iota_{l}$. For the decreasing curve this time corresponds to the interval [0.95; 1.05]*$L_{2\_low}$ and is referred to as $\iota_{h}$.

(2) $$\xi_{l}=\frac{\left(l_{1\_low\_f}-L_{1\_low}\right)}{\left(t_{1\_low\_f}-t_{1\_low\_i}\right)}*100\%$$

With $l_{1\_low\_f}$, $L_{1\_low}$, $t_{1\_low\_f}$ and $t_{1\_low\_i}$ parameters defined in Figure 7D. This equation is also valid for any interval of time immediately after turning on the voltage with $l_{1\_high\_f}$, $L_{1\_high}$, $t_{1\_high\_f}$ and $t_{1\_high\_i}$.

An example of closing specimen is presented in Figure 7E. The left image proposes a photo taken without voltage in the specimen. The middle image is a photo taken short time after voltage is switched on. This photo also displays an example of the tracker points used to obtain the width of one the ten actuators. Finally the right image presents a photo taken as the specimens are converging toward their closed position when the voltage is applied. The degree of closing is calculated from the images acquired during the experiment. The actuation strain is used to quantify the degree of closing of the specimens. Each acquired image is split between upper (top in Figure 7E) and the lower (bottom in Figure 7E) parts to separate the two specimens. Each part of the image is dissociated in five actuating parts treated separately. The image section of each actuator is assigned with tracking points using a Python script. To track points in space the OpenCV function *goodFeaturesToTrack* was used with *maxCorners* equal to 20 000 and a *blockSize* of 7. One of this actuating part and its tracking points are presented in Figure 7E. Then these points had their position tracked during the experiment. The actuation width *w* of the square containing all these point was measured along the experiment.

The actuation strain (representing the evolution of the actuation width *w* defined in Figure 7E is measured for each actuator with equation 3.

(3) $$\varepsilon =\frac{\left(w_{0}-w_{i}\right)}{w_{0}}*100\%$$

In Equation (3), $w_{i}$ represents the width of the actuator as displayed in Figure 7E at a given time *i*. The parameter $w_{0}$ is the width measured on on the first image taken. The average is calculated from the five actuators of the specimen.

### 4.4 Individual Behavior of Material Actuating Neurone

To assess the voltage‐input/structure‐response relationship, a separate experiment varied the voltage in the second actuator (from the top, Figure 2B while keeping all other specimens unpowered. Figure 2C also shows the measured output light and strain for each of the four actuators of the specimen considered in the QSDF aperture for this test. Equation (3) is used to calculate the strain as a representation of the degree of opening [5].

### 4.5 Control of the PAMMUNN via Data‐Aware Back‐Propagation Training

**FIGURE 8:**
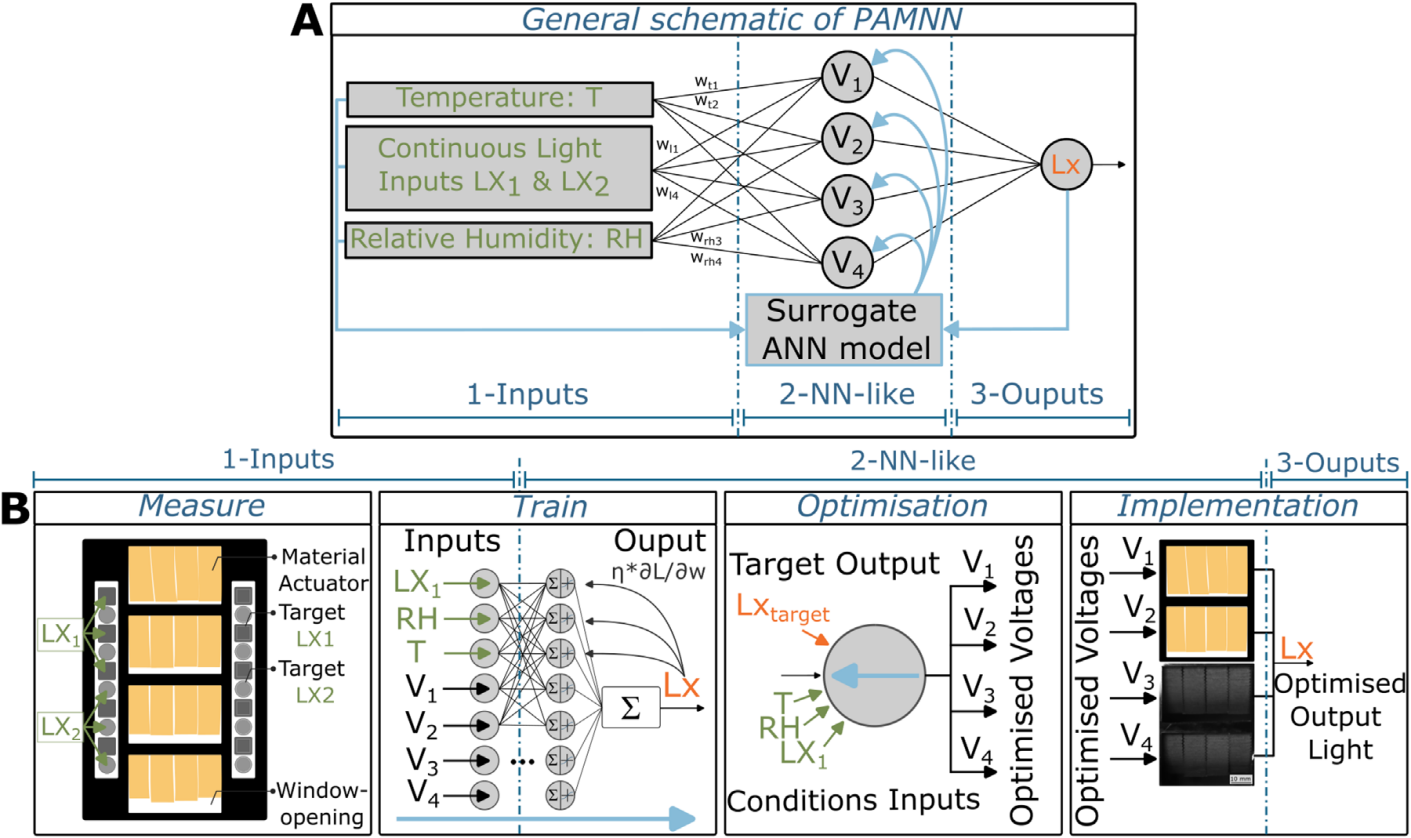
Schematics of functioning of the artificial neural network‐controlled structure via Data‐Aware Back‐Propagation Training. (A) General schematic presenting the links between inputs (temperature, light outside, relative humidity) and the output (Light inside the box). (B) Detailed schematics of the window‐like structure and the calculations performed for its control. *Measure*: view of the actuator from the camera, showing how the light targets used to measure ${\mathrm{LX}}_{1}$ and ${\mathrm{LX}}_{2}$ are positioned. *Train*: Schematics for the training process of the neural network linking output and input. *Optimization*: schematics of the optimization process to obtain voltage from the target light and the conditions of the system. *Implementation*: Use of optimized voltages from the experimental set up to reach the desired output light.

Figure 8 presents the logic behind the control of the window‐like structure via artificial neural network. The structure controlled is made of four actuating specimen placed one above the other, as the schematic of QSDF aperture presented in Figure 2B presents.

**FIGURE 9:**
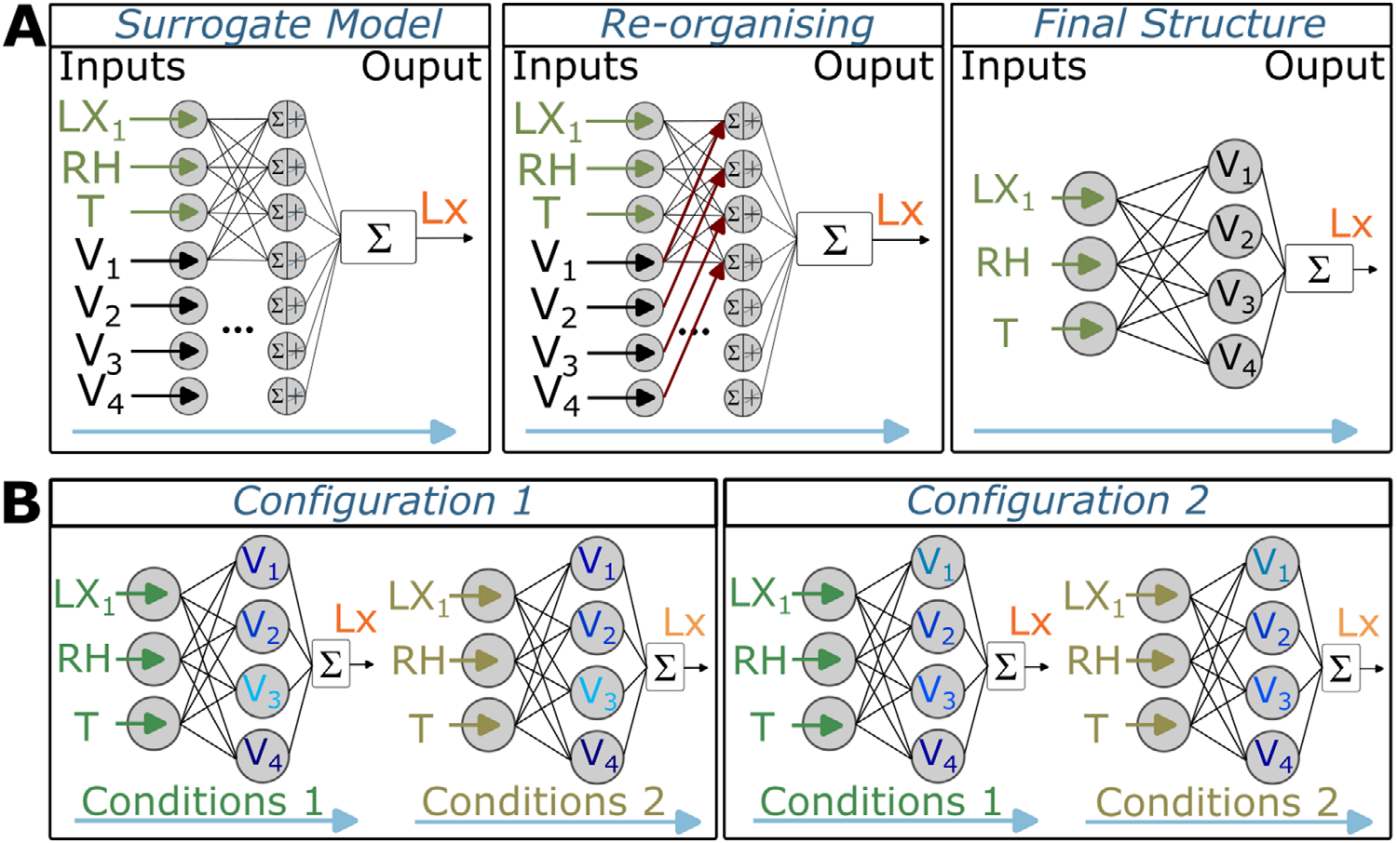
Schematics of functioning of the artificial neural network‐controlled structure via Data‐Aware Back‐Propagation Training and schematic of the creation of the physical‐mode. (A) Schematic of the optimizer re organization to obtain PAMMUNN‐optimized configuration. (B) Schematic of the two configurations selected to observe the behavior of the PAMMUNN.

Figure 8A describes the target behavior for the control of the window made with the physical adaptive material neural network. This figure illustrates that the physical nature of the inputs leads their quantities arriving on each specimen to be variable. For instance, heated air tends to rise as a result of density. Hence, $w_{t1}$, $w_{t2}$ are the weight coefficients representing this variation between the temperature received by each specimen and the general input temperature value considered for the control of the machine. Measuring exact temperature, humidity and light going to each actuating specimen is complex. Therefore, the approach implemented here is to measure a general value for each of the input parameter and observe the ability of the control strategy to account for these variations without precisely measuring them. The first step in implementation of the NN‐like window structure is training (Figure 8B). During this step, a database is created by measuring the output light in the light sensor in Figure 2C and linking it to the environmental parameters (input light, relative humidity and the temperature). The ${\mathrm{LX}}_{1}$ and ${\mathrm{LX}}_{2}$ light input parameters are considered, representing the RGB colour coefficients of gray targets positioned a few millimetres beside the NN‐like window. This acquisition method was selected for its high repeatability and minimal impact on light transmission through the window. Figure 8B shows the position of the light targets used for measuring ${\mathrm{LX}}_{1}$ and ${\mathrm{LX}}_{2}$. The square shape target are used to measure ${\mathrm{LX}}_{1}$ and are filled with dark grey (i.e., RGB values [98; 98; 98]). The circle shape targets are used to measure ${\mathrm{LX}}_{2}$ and are filled with a light grey (i.e., RGB values [148; 148; 148]). The positioning of the camera for the acquisition of images is shown in Figure 2C. The images are then uploaded in a Python script, as could be seen in Figure 8B a white circle is plotted in each circle target and a white square is drawn in each square light targets. The greyscale value of the pixel in the plotted circles and squares are then measured and averaged to obtain the value of ${\mathrm{LX}}_{1}$ and ${\mathrm{LX}}_{2}$, respectively. For the data selection the main criterion was to cover a wide scope of light inputs to represent different solar configurations in potential future applications. Figure 3B presents an example of the database used for training. The final version of the training database has a coefficient of variation of 39% for the input light. This value indicates a relatively high spread in the data. The data were intentionally sampled with a bias as it would be the case for the weather in a given environment. Accordingly, this bias is highlighted by the difference between the mean and the median representing 4% of the range of the data used. This highlights a bias toward smaller values of light input. This is consistent with the fact that during the day the light intensity is at its highest at midday and lower at both dawn and dusk. The remaining data points were collected while the system responded to variations in the input light. Therefore, all predictions conducted and presented in this study were made with a different database. During machine operation, 13 data points were considered as faulty and manually deleted from the working database. The main reasons for the data points being suppressed include non‐convergence and instability of the output light signal before saving the data, asynchrony between the different parameters recorded (highlighted in Figure 2) during machine operation, and experimental issues during operation. Some of these issues were observed after the data was used for optimization and prediction, demonstrating robustness of the machine which functions despite faulty data points. The database is used to train a single‐layer neural network with seven nodes and a sigmoid activation function (Figure 9A). A gradient descent optimization algorithm with a η = 0.54 learning rate is used to adjust the weight of the NN layer. This value was selected after an initial optimization of the hyperparameters of the study was conducted. The loss function for the training is calculated as $L=\frac{1}{2}*{\left(LX-LX_{target}\right)}^{2}$. In the training first the inputs and output were scale between 0 and 1. For the training, the ${\mathrm{LX}}_{1}$ values were considered as input and the ${\mathrm{LX}}_{2}$ were used to verify the quality of the ${\mathrm{LX}}_{1}$ values obtained. As described in Figure 8B *Sigmoid* functions are used for the layer of nodes. The database obtained was divided in 80% and 20% for training and validation, respectively. To avoid overfitting data, the training was stopped after 2000 epochs if no improvement on the validation loss was achieved. In the training process described in Figure 8B, the voltages used to obtain the database and optimized range varied between 0.0 and 35.0 V. As described in the Optimization section of Figure 8B, the neural network structure was used as a surrogate model to predict output light. This approach described in Figure 8B uses the *optimise* (method = “*L‐BFGS‐B*”) function from the *Scipy* package in Python is used to minimise the square of the difference between targeted light and the one obtained with a feed forward calculation using the weights determined during the NN training. The voltages derived are then implemented to achieve the desired light output specified by the user. This method is inspired by Physics‐aware back propagation training used in PNN which requires a digital model to predict the behavior of the system and optimized the controlling parameter [23]. Hence, we call data‐aware back propagation training the method used here as it does not require a digital model but a database to predict the behavior of the PAMMUNN machine. To limit the energy consumed and speed up the actuation, 30 optimized solutions have been identified for the voltages, and the solutions with the lowest sum between the four voltages was selected and implemented. In the Implementation section, the two bottom photos show the actuators closing as voltage is applied to their carbon black composite sections.

The database was acquired and its size was increased after each run by collecting data during the test presented in discussion. Liu et al. highlighted that 35.8% of the datasets they observed in material science studies using data‐driven machine learning have a ratio of the number of features to the sample size greater than 0.25 [43]. This ratio is associated with the problem of high dimensionality in small datasets. To avoid such an issue in the present study the feature to sample ratios used ranged from 0.023 to 0.019. This helps the surrogate model avoid issues associated with small datasets. No less than 300 data points were used for the database used for the training of the neural networks presented in this study. Optimized voltage values were then obtained to reach desired output. The voltage outputs from the setup influence both the relative humidity and the temperature of the system. Therefore, real‐time humidity and temperature readings cannot be used directly in the optimization, as they do not reflect the steady‐state conditions at the optimal operating point. To calculate the optimal voltage outputs, it is necessary to estimate the steady‐state temperature and humidity values. In order to achieve this, the coefficients of determination between the voltage values and the temperature and humidity were obtained with the Least Squares regression Python *np.polyfit* function for the database of 300 data points (functions provided in Supporting Information). Initial voltage optimization was then performed. Using the coefficients of determination, we then estimated the stabilized temperature and relative humidity at convergence. These updated values were reintroduced into the optimization code for a second, refined voltage optimization run. The values of voltage obtained were introduced again in the regression function to obtain the final predicted values of temperature and humidity to be used on the final prediction round with the trained Neural Network. A weaker correlation between voltage and temperature than between voltage and humidity was observed empirically. Hence, following trials the adjusted the current temperature had only 10% of the difference between the predicted and current temperatures was added to the adjusted current temperature value used in the calculation. This value serves as an experimental learning rate to obtain an approximation of the temperature. When the optimized voltages were calculated, an overshoot was sent to the voltage to accelerate the convergence to the targeted interval. For each voltage, the difference between the current and the optimized values was calculated. To reach the target interval, 105% (95% for V4 in Figure 8B) of this difference was added to the optimized value for decreasing output light, and 180% for increasing output light. These values were obtained after empirical trials to optimize the actuators convergence speed to their final shape. They were also selected to not influence the final machine output. Once the target interval was reached with the overshoot voltage values, the system applied the optimized voltage values to conclude the convergence of the output light.

### 4.6 PAMMUNN: Incremental Learning

For the control system shown in Figure 8, adaptability to its environment is crucial. van de Ven et al. define incremental learning as a system learning from a non‐stationary stream of data [44]. The system described in Figure 8 acquires new data by letting it evolve within its environment. For physical neural networks, database creation is time‐dependent, which in turn affects the training process [23]. This test aims to demonstrate that the machine enhances its predictive accuracy over time by adapting its data input stream. In this study, learning involves exploring the design space to accommodate a wider range of conditions. To assess the system's learning capability, the neural network's predictive accuracy was evaluated based on the volume of acquired data. To achieve this, 35 points/configurations have been initially randomly selected from the database for evaluation. The model has been then trained with increasing numbers of data points, starting from five then increasing by five until a database of 80 points. The interval of increase of the database has been then adjusted to 20 points for every evaluation, until the entire database was added for training out of the 35 points initially extracted for evaluation.

### 4.7 PAMMUNN: Physical‐Mode

In order to build a physical adaptive material neural network with less digitally dependent, the surrogate model should be removed from the control of the actuator during operation. This mode of operation, relying less on digital apparatus it is named *Physical‐mode*. Figure 9A illustrates the configuration transition from the surrogate model‐controlled structure discussed earlier to the physical‐mode. Inspired by Lee et al. [25], who demonstrated mechanical neural network capabilities, these tests will identify two distinct configurations capable of producing identical outputs. Figure 9B presents a schematic of neural networks related to two configurations of specimen with voltages assigned. Each configuration is capable of obtaining the same outputs (one orange nuance for each output) when given a similar set of inputs (one green nuance for each input). To achieve the required configurations, Lee et al. modeled the mechanical neural network and then run an optimization on the model. To achieve Configurations 1 and 2 (Figure 9B), we applied the surrogate model optimization technique shown in Figure 9B. The neural network underwent identical training, with optimization focused on minimizing the sum of squared differences between the targeted and optimized light outputs under both conditions. The calculated voltage values were accepted only if the difference between individual voltages exceeded 3 V. For instance, in the example provided in Figure 9B the conditions targeted are: Conditions 1: (Lx: 180.3 Lx; ${\mathrm{LX}}_{1}$: 185; T: 21.9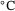; RH: 91.0%) Conditions 2: (Lx: 115 Lx; ${\mathrm{LX}}_{1}$: 110.44; T: 22.3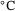; RH: 91.5%). The optimization allowed to obtain [0.23; 20.42; 0.01; 8.34] V and [23.32; 14.01; 6.06; 14.10] V for the two configuration making the prediction of light output (condition 1: 181.2 LX and 114.0 LX) and (condition 2: 183.4 LX and 110.1 LX), respectively. These testing parameter were the implemented in the QSDF set up presented in Figure 2 with the objective to validate the ability of the machine created to behave in the physical‐mode.

## Conflicts of Interest

The authors declare no conflicts of interest.

## Supporting information




**Supporting File**: advs77473‐sup‐0001‐SuppMat.zip.

## Data Availability

The data that support the findings of this study are available from the corresponding author upon reasonable request.
